# Supplementary material for: Topological solitonic macromolecules
Source: Nat Commun. 2023 Jul 29;14:4581. doi: 10.1038/s41467-023-40335-5 (PMC10387112; doi:10.1038/s41467-023-40335-5)
Supplement: Supplementary file 1 — Supplementary Information [file 41467_2023_40335_MOESM1_ESM.pdf]

Supplementary Information

Topological solitonic macromolecules

Hanqing Zhao[1], Boris A. Malomed[2,3] and Ivan I. Smalyukh[1,4,5,6*]

[1]Department of Physics, University of Colorado, Boulder, CO 80309, USA

[2]Department of Physical Electronics, School of Electrical Engineering, Faculty of Engineering, and Center for Light-Matter Interaction, Tel Aviv University, P.O.B. 39040, Ramat Aviv, Tel Aviv, Israel

[3]Instituto de Alta Investigación, Universidad de Tarapacá, Casilla 7D, Arica, Chile

[4]Materials Science and Engineering Program, University of Colorado, Boulder, CO 80309, USA

[5]International Institute for Sustainability with Knotted Chiral Meta Matter, Hiroshima University, Higashi Hiroshima, Hiroshima 739-8526, Japan

[6]Renewable and Sustainable Energy Institute, National Renewable Energy Laboratory and University of Colorado, Boulder, CO 80309, USA

* *Correspondence to: ivan.smalyukh@colorado.edu*

Supplementary Figures and Captions

Letter	ASCII code	Skyrmion number	Preimage
A	$(01000001)_2$		
B	$(01000010)_2$		
C	$(01000011)_2$		
D	$(01000100)_2$		
E	$(01000101)_2$		
F	$(01000110)_2$		
G	$(01000111)_2$		
H	$(01001000)_2$		
I	$(01001001)_2$		
J	$(01001010)_2$		
K	$(01001011)_2$		
L	$(01001100)_2$		
M	$(01001101)_2$		
N	$(01001110)_2$		
O	$(01001111)_2$		
P	$(01010000)_2$		
Q	$(01010001)_2$		
R	$(01010010)_2$		
S	$(01010011)_2$		
T	$(01010100)_2$		
U	$(01010101)_2$		
V	$(01010110)_2$		
W	$(01010111)_2$		
X	$(01011000)_2$		
Y	$(01011001)_2$		
Z	$(01011010)_2$		

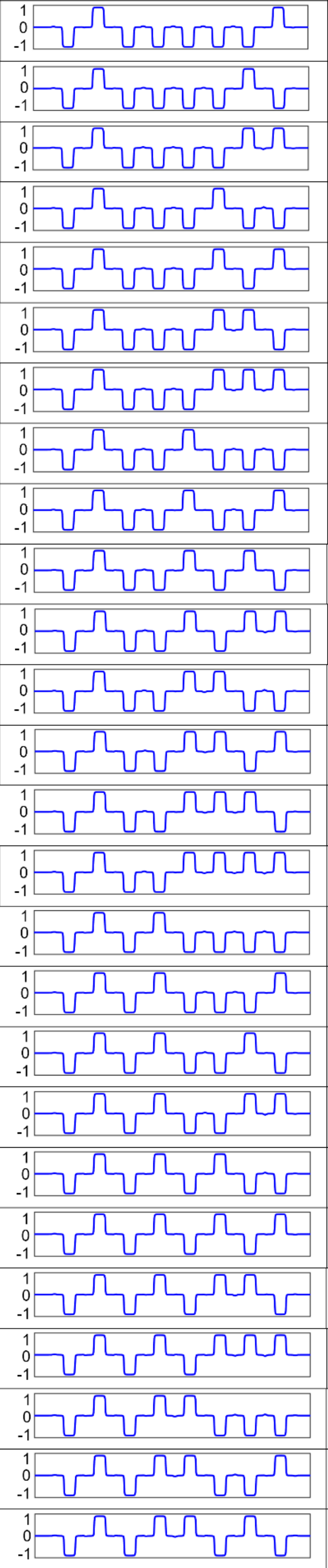

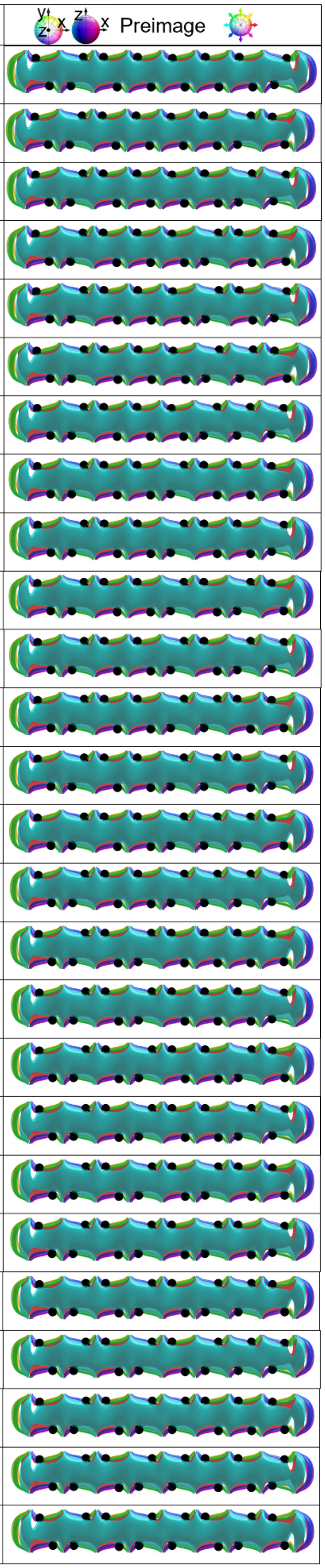

Supplementary Fig. 1| Encoding alphabet with polyskyrmionomers and ASCII binary codes. Defects in the middle of the structures, which affect the skyrmion number symbol, are marked by black sphere. The colour code used in the preimages and the selected orientations of preimages are marked on the top-right.

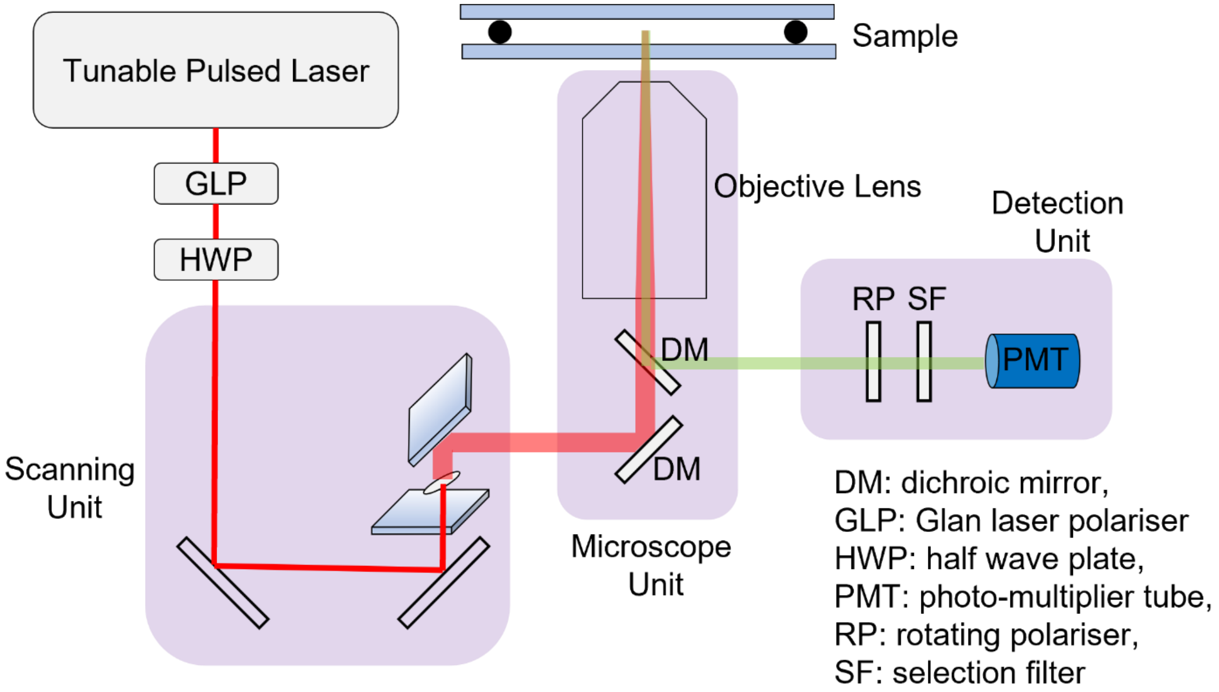

Supplementary Fig. 2| A schematic diagram of the 3PEF-PM imaging setup. The scanning excitation laser light travels along the red path, whereas the emitted fluorescence light collected with the objective in the epi-detection geometry follows the green path.

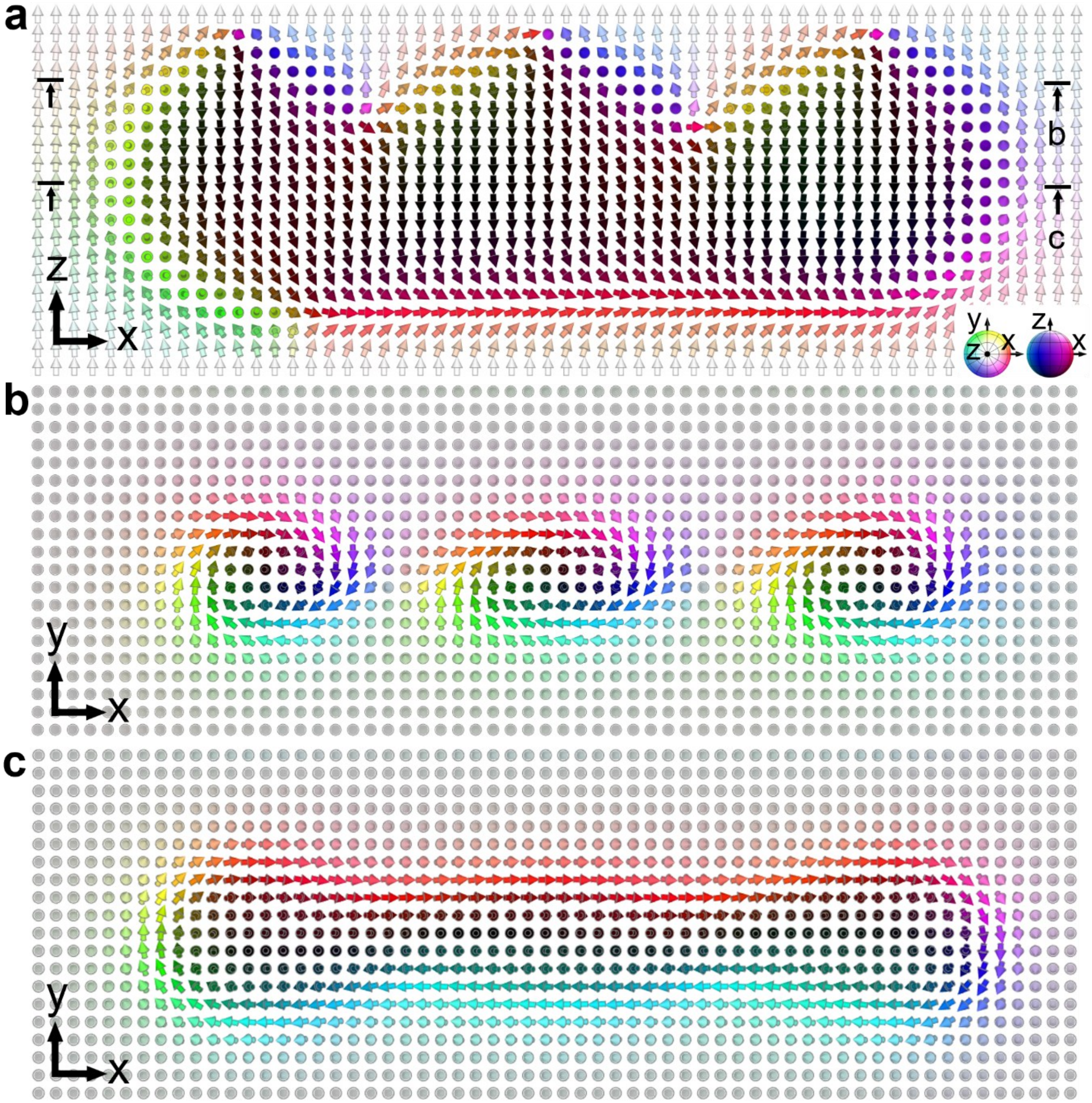

Supplementary Fig. 3| Vectorized director field of the $\mathbf{S_1^3}$ in different cross-sections. a, The (*x*,*z*) cross-section in midplane along the *y* direction. **b**,**c**, The top quarter plane (**b**) shows three skyrmions, and the midplane (**c**) shows a single distorted one. The coloured arrows correlate the smoothly vectorized orientations of **n**(**r**) with points on the two-sphere order parameter space (**a**).

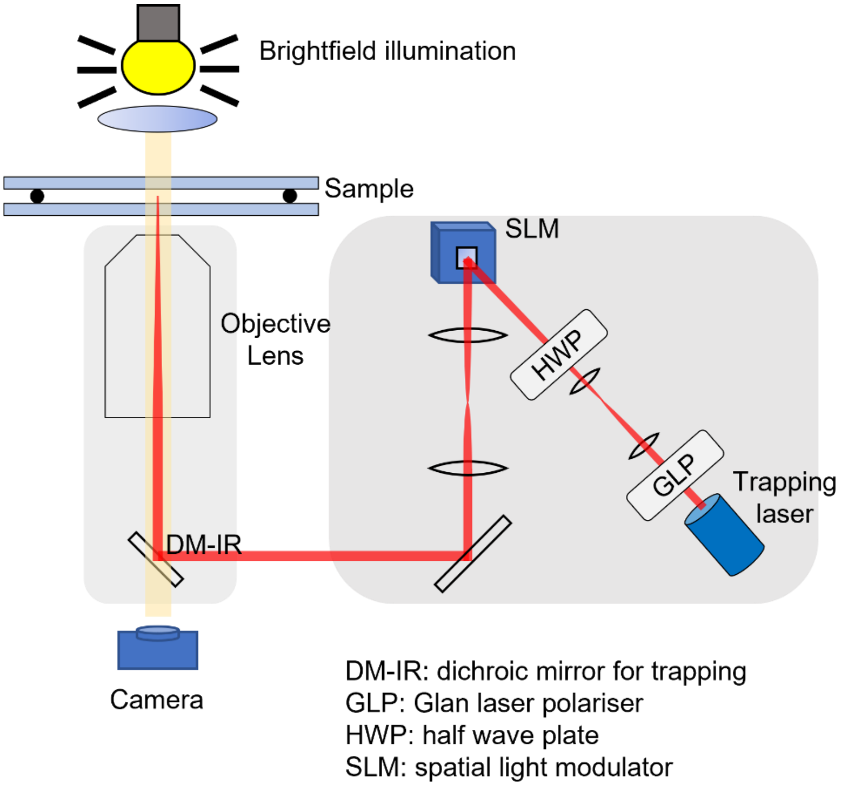

Supplementary Fig. 4| A schematic diagram of the laser trapping setup. The trapping laser light travels along the red path, and the imaging light follows the yellow path.

Details of 3PEF-PM imaging

The three-photon excitation fluorescence polarizing microscopy (3PEF-PM) imaging technique is based on nonlinear light-matter interaction and the nonlinear optical response of 5CB component. In our experiments, it is a powerful tool for non-invasive, label-free imaging with high three-dimensional (3D) resolution. Three-photon excitation is a nonlinear process, which occurs within a small volume corresponding to the focus of the infrared femtosecond laser light, leading to the absorption of three photons of near-infrared light and the ensuing emission a single photon at a visible-range wavelength much shorter than the excitation light. In addition, we obtain the 3D and various cross-sectional images for different polarizations of the excitation light by using half-wave plates. A simple schematic diagram of 3PEF-PM setup is shown in the Supplementary Fig. 2. The setup utilizes a tunable pulsed laser, 3D scanning mirrors, an inverted microscope with photo-multiplier tube (IX-81 Olympus inverted microscope), and a rotating polarizer. When we scan the sample in 3D, the laser focal point in the (x,y) plane is controlled by scanning mirrors (the scanning unit in Supplementary Fig. 2) and the vertical position of the focal point is adjusted with a stepper motor (10-nm precision).

Details of the laser trapping setup

The laser manipulation setup utilizes a reflective, electrically addressed, phase-only spatial light modulator (P512-1064, Boulder Nonlinear Systems) with 512×512 pixels (15×15 μm^2 in size) and an ytterbium-doped fibre laser (YLR-10-1064, IPG Photonics) operating at 1064 nm. A simple schematic diagram of the laser trapping setup is shown in the Supplementary Fig. 4. The

polarization of the trapping beam is controlled by a Glan-laser polarizer and a half-wave retardation plate. The spatial light modulator controls the phase of the beam on a pixel-by-pixel basis, using computer-generated holograms (at a refresh rate of 30 Hz). The beam is then imaged at the back aperture of the microscope objective. When trapping polyskyrmionomers in the stretching mode (Supplementary Movie 4), we split the laser beam into two beams and optically trap both ends of the polyskyrmionomer. We then stretch the polyskyrmionomer to a certain distance and turn off the laser trapping, allowing the extended polyskyrmionomer to relax back to the equilibrium length. For the characterization of the scissoring mode (Supplementary Movie 4), we split the laser beam into three beams and trap the two ends and centre first, move the central beam away from the equilibrium position vertically and then remove that trapping laser beam; following this, the structure relaxes back to the initial configuration, which we characterize.
