# Supplementary material for: Topological solitonic macromolecules
Source: Nat Commun. 2023 Jul 29;14:4581. doi: 10.1038/s41467-023-40335-5 (PMC10387112; doi:10.1038/s41467-023-40335-5)
Supplement: Supplementary file 3 — Description of Additional Supplementary Files [file 41467_2023_40335_MOESM3_ESM.docx]

File Name: Supplementary Movie 1

Description: Experimentally-reconstructed (left) and computer-simulated (right) structures of $S_{1}^{2}$ and $S_{1}^{3}$. $S_{1}^{2}$ (top) and $S_{1}^{3}$ (bottom) modes are shown by means of $\left| n_{z} \right|$= 0.8 isosurfaces coloured according to local orientations of the director, in the same way as it was done in Fig. 4a.

File Name: Supplementary Movie 2

Description: Self-propelling rotation of $S_{1}^{2}$ (top left), $S_{2}^{1}$ (top mid), $S_{1}^{3}$ (top right) and order-17 polyskyrmionomers (bottom). The movies are obtained for cell gap *d* =10 μm, *d/p* = 2, *U*_0_ = 3.9 V and *f* = 100 Hz. The orientations of the crossed polarizer and analyser, elapsed time and scale bar are marked on movie frames.

File Name: Supplementary Movie 3

Description: Brightfield transmission-mode optical movie showing the breathing dynamics of a trimer under the action of the modulating electric field. The movie is produced for the LC cell gap *d* =10 μm, *d/p* = 2, *U*_0_ = 3.85 V, Δ*U* = 0.05 V, *f* = 3000 Hz and the modulating frequency 1 Hz. The elapsed time and scale bar are marked on movie frames.

File Name: Supplementary Movie 4

Description: POM movies showing stretching (top) and scissoring (bottom) overdamped vibration of trimers. For the stretching overdamped vibration (top), the laser tweezers trap the two ends initially and drag the trimer away from the state with the equilibrium length, then we switch off the laser tweezers and let the trimer relax. For the scissoring overdamped vibration (bottom), the laser tweezers trap the two ends and the centre initially, and first drag the centre away from its equilibrium position. To show the evolution in the vertical direction, we switch off the centre-trapping laser beam of the tweezer system and then let the trimer relax. The movie is produced with cell gap *d* =10 μm, *d/p* = 2, *U*_0_ = 3.9 V and *f* = 3000 Hz. The orientations of the crossed polarizer and analyser, elapsed time and scale bar are marked on movie frames.

File Name: Supplementary Movie 5

Description: POM movies showing two interacting dimers (top left), an electrically activated dimer array (top mid) and dimer gas (top right), co-propulsion of a möbiuson and dimer (bottom left), a toron interacting with the activated dimer gas (bottom mid), and a hopfion interacting with activated dimers’ gas (bottom right). The movies are produced for the LC cell gap *d* =10 μm, *d/p* = 2, *U*_0_ = 3.9 V and *f* = 100 Hz. The orientations of the crossed polarizer and analyser, elapsed time and scale bar are marked on movie frames.

File Name: Supplementary Movie 6

Description: The self-propelling motion of the star-shaped trimer. The movies are produced for cell gap *d* =10 μm, *d/p* = 2, *U*_0_ = 3.9 V and *f* = 100 Hz. The orientations of the crossed polarizer and analyser, elapsed time and scale bar are marked on movie frames.

File Name: Supplementary Movie 7

Description: POM movies showing the order-annihilation process for a pentamer, as seen from the experiment (top) and predicted by the simulations (bottom). The POM movies are produced for the LC cell gap *d* = 10 μm, *U*_0_ = 4.0 V and *d/p* = 2. The orientations of the crossed polarizer and analyser, elapsed time and scale bar are marked on movie frames.

File Name: Supplementary Movie 8

Description: The transformation of a polyskyrmionomer from the ring configuration to a linear one. The movie is produced for cell gap *d* =10 μm, *d/p* = 2 and *f* = 3000 Hz. The orientations of the crossed polarizer and analyser, elapsed time and scale bar are marked on movie frames.

File Name: Supplementary Movie 9

Description: Self-folding of an order-18 polyskyrmionomer. The movie is produced for the LC cell gap *d* =10 μm, *d/p* = 2, *U*_0_ = 3.9 V and *f* = 100 Hz. The orientations of the crossed polarizer and analyser, elapsed time and scale bar are marked on the movie frames.
